# Macrophage pro-inflammatory cytokine secretion is enhanced following interaction with autologous platelets

**DOI:** 10.1186/1476-9255-7-53

**Published:** 2010-11-11

**Authors:** Christopher M Scull, William D Hays, Thomas H Fischer

**Affiliations:** 1 Francis Owen Blood Research Lab, Department of Pathology and Laboratory Medicine, University of North Carolina at Chapel Hill, 125 University Lake Rd, Chapel Hill, NC 27516, USA

## Abstract

**Background:**

Macrophages are the dominant phagocyte at sites of wound healing and inflammation, and the cellular and acellular debris encountered by macrophages can have profound effects on their inflammatory profile. Following interaction with apoptotic cells, macrophages are known to switch to an anti-inflammatory phenotype. Activated platelets, however, are also a major component of inflammatory lesions and have been proposed to be pro-inflammatory mediators. In the present study, we tested the hypothesis that macrophage interaction with activated platelets results in an inflammatory response that differs from the response following phagocytosis of apoptotic cells.

**Methods:**

Human monocyte-derived macrophages (hMDMs) were co-incubated with autologous activated platelets (AAPs) and the platelet-macrophage interaction was examined by electron microscopy and flow cytometry. The cytokines TNF-α, IL-6, and IL-23 were also measured during LPS-activated hMDM co-incubation with AAPs, which was compared to co-incubation with apoptotic lymphocytes. Cytokine secretion was also compared to platelets pre-treated with the gluococorticoid dexamethasone.

**Results:**

Macrophages trapped and phagocytized AAPs utilizing a mechanism that was significantly inhibited by the scavenger receptor ligand fucoidan. LPS-induced macrophage secretion of TNF-α, IL-6, and IL-23 was inhibited by co-incubation with apoptotic cells, but enhanced by co-incubation with AAPs. The platelet-dependent enhancement of LPS-induced cytokines could be reversed by pre-loading the platelets with the glucocorticoid dexamethasone.

**Conclusions:**

The interaction of human macrophages with autologous platelets results in scavenger-receptor-mediated platelet uptake and enhancement of LPS-induced cytokines. Therefore, the presence of activated platelets at sites of inflammation may exacerbate pro-inflammatory macrophage activation. The possibility of reversing macrophage activation with dexamethasone-loaded platelets is a promising therapeutic approach to treating unresolved inflammation.

## Background

A major function of macrophages is phagocytosis of cellular and acellular debris during inflammation and wound healing, and the activation response of macrophages following phagocytosis can be varied depending on the local extracellular environment [1-6]. The importance of phagocytosis in the resolution of inflammation is emphasized by pathological conditions involving impaired phagocytosis, which may manifest as persistent infections or chronic inflammatory lesions such as diabetic ulcers and atherosclerotic plaques [7-11].

During their differentiation from primary monocytes, macrophages acquire specialized receptors and machinery for recognizing and clearing both apoptotic and infected cells [12]. In clearing apoptotic cells, macrophages use receptors such as scavenger receptors and integrins that function independently or in cooperation with each other depending on the type of cell targeted for phagocytosis [13-19].

Platelets are anucleate cells which play an integral role in maintaining vascular integrity. Within their 8-10 day lifespan, platelets can become activated either in the circulation or during adherence at a site of injury, and during this process they become targeted for destruction by macrophages [20]. The process of platelet activation involves several changes to the cell surface, including expression of P-selectin and loss of membrane asymmetry [21-23]. These changes in the platelet membrane may provide molecular signals to macrophages that trigger phagocytosis, although the precise mechanism by which macrophages recognize and phagocytose activated platelets remains to be identified.

Cells that have become apoptotic as part of their normal life cycle are recognized and cleared by phagocytosis in a manner that usually inhibits pro-inflammatory responses [24-27]. Although circulating platelets can exert a pro-inflammatory effect on circulating monocytes [28,29], their effect on differentiated macrophages, particularly at sites of inflammation, is not clear. We show here that phagocytosis of autologous platelets results in an pro-inflammatory profile that is opposite to the macrophage response following phagocytosis of apoptotic cells. Importantly, the platelet-enhanced, pro-inflammatory response of macrophages can be inhibited when the platelets are loaded with the glucocorticoid dexamethasone. In addition to novel insight into the macrophage inflammatory profile that exists in several diseases, these results also provide evidence that platelet-macrophage interactions are an important therapeutic target for reducing inflammation.

## Methods

### Monocyte-derived Macrophages

Human monocytes were isolated and cultured using techniques similar to those previously described [30,31]. Briefly, blood from healthy human donors was collected into citrate and peripheral blood mononuclear cells (PBMCs) were isolated by using Lymphoprep (Accurate Chemical) according to the manufacturer's instructions. Monocytes were further isolated by plating the PBMCs on gelatin-coated tissue culture flasks for 45 min at 37°C followed by 10 washes with phosphate buffered saline (PBS) to remove non-adherent lymphocytes. Monocytes were then detached from the flasks by incubation in 10 mM EDTA for 2 min at 37°C. Monocytes (250,000 in 500 μl volume) were then plated in 24-well plates overnight in RPMI 1640 supplemented with 10% fetal bovine serum (FBS) and 10 ng/ml recombinant human GM-CSF (R&D Systems). Monocytes were plated on glass coverslips for scanning electron microscopy (SEM) analysis and plastic tissue-culture plates for TEM analysis and phagocytosis experiments. Media was changed on day 2 and day 5, and after 7 days of culture the MDMs were used for phagocytosis and cytokine assays.

### Platelets

Platelets were isolated from whole blood collected into acid-citrate-dextrose (ACD) from healthy human donors and centrifuged for 15 min at 500 × *g *to generate platelet-rich plasma (PRP). PRP was pelleted by centrifugation for 10 min at 800 × *g *and the platelet pellet subsequently washed 2 times in citrated saline (pH 6.8). A portion of the platelet samples were degranulated by incubating 1 ml of platelets (250,000/μl in citrated saline) with 10 μl of 10 μM calcium ionophore A21387 (Sigma) for 15 min with rocking at room temperature, followed by three centrifugational washes with citrated saline. For phagocytosis experiments, platelets were fluorescently labeled with Cell Tracker Green CMFDA (Invitrogen) as previously described [32]. After the final wash, and prior to use in phagocytosis assays, platelets were resuspended in warm serum-free RPMI for 15 min at 37°C.

For analysis of surface P-selectin and phosphatidylserine, platelets (250,000/μl) were first incubated in either citrated saline or serum-free RMPI media for 1 hr at 37°C. A portion of the platelets were activated by including thrombin (King Pharmaceuticals, 1 U/ml final concentration) in the incubation reaction. To detect surface expression of activation markers in each platelet treatment group, a 10 μl aliquot of platelets was stained with either FITC-anti-CD62P (Biolegend) or FITC-Annexin-V (Biolegend) for 30 min at room temperature, after which the cells were fixed and analyzed immediately by flow cytometry. Flow cytometry analysis was performed using a CyAn flow cytometer (Beckman Coulter) and the Summit analysis software.

Dexamethasone-loaded platelets were prepared by incubating 1 ml of platelets (250,000/ul) in citrated saline with 5 ul of dexamethasone (Sigma, 10 mM in DMSO) for 15 min on a rocker at room temperature. Platelets were then washed three times with citrated saline to remove unbound dexamethasone. For subsequent experiments, 5 × 10^6 ^dex-platelets were added to each well of macrophages in a 24-well plate.

To prepare apoptotic cells, PBMCs were isolated as above, and following monocyte-adherence to gelatin-coated flasks the non-adherent lymphocytes were collected. Cells were rendered apoptotic (Annexin-V positive) by UV-irradiation for 10 min followed by overnight incubation in RPMI + 10% FBS at 37°C + 5% CO2.

### Phagocytosis Experiments

Thirty minutes prior to the start of each experiment, 7-day old MDMs were washed 3 times with PBS and incubated with 500 μl fresh RPMI media. In some experiments the media was supplemented with 10% autologous human serum. A 25 μl aliquot of fluorescently labeled platelets (250,000/μl) was added to each well of macrophages. Platelets and macrophages were co-incubated for 45 min.

For SEM analysis, the co-cultures were washed once with PBS and fixed in 2% paraformaldehyde and 0.5% glutaraldehyde. The samples were then processed as previously described [33] and examined using a Cambridge S200 scanning electron microscope at 20 kV. For TEM analysis, the co-cultures were washed 5 times with PBS and fixed in 2% paraformaldehyde, processed as previously described [34], and examined using a Leo EM 910 transmission electron microscope. For flow cytometric analysis, warm trypsin/EDTA was then added to the macrophages to remove adherent platelets (confirmed by microscopy) and cells were incubated 15 min at 37°C. Macrophages were then collected and fixed in 1% cold paraformaldehyde and analyzed using a CyAn flow cytometer (Beckman Coulter) and the Summit analysis software. Data is expressed as the percentage of FL1-positive macrophages in a given collection of 10,000 macrophages. Data shown represent the average of at least 3 independent experiments and for each experiment 10,000 macrophages were analyzed.

Latrunculin (Sigma, 1 μg/ml final concentration), used as a pan-phagocytosis inhibitor, was added to a portion of macrophage-containing wells 30 min prior to addition of platelets. Fucoidan (Sigma) was added to macrophages at a final concentration of 250 μg/ml 30 min prior to addition of platelets.

### Cytokine Experiments

Each well of MDMs was washed 3 times with PBS and incubated with fresh RPMI + 10% autologous human serum. Activated, degranulated, or dexamethasone-loaded platelets (5 × 10^6^) were added to each well in addition to LPS (100 ng/ml). Some samples also received dexamethasone alone at a final concentration of 1 μM. After 24 hrs, supernatants were collected, spun 10 min at 14,000 g, and frozen at -80°C. Cytokines were measured by ELISA using capture and detection antibodies (eBioscience) per the manufacturer's instructions. Cytokines were measured in duplicate and averaged. The amount of protein secreted was normalized to the amount secreted by macrophages treated with LPS alone. Each experiment was performed at least 3 times using 3 different MDM donors. In each experiment, the platelets added were from the same donor as the MDMs. Treatment groups were compared using an unpaired t-test.

## Results

### Macrophage Phagocytosis of Autologous Platelets

**Figure 1:**
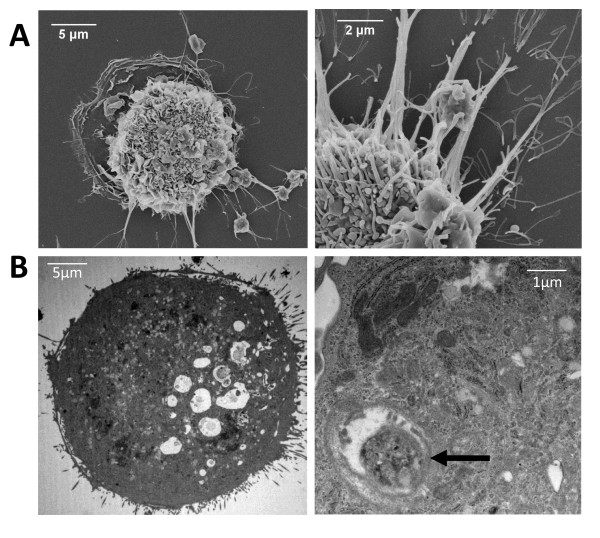
**Electron Microscopic Analysis of Platelet-Macrophage Interactions**. **(A) **hMDMs were incubated with fresh AAPs for 15 min (left panel) or 45 min (right panel) before processing for SEM analysis. **(B) **TEM analysis of hMDMs after 1 hr co-incubation with platelets. Arrow indicates phagocytic vacuole at high magnification.

To examine the interaction between human MDMs (hMDMs) and autologous platelets, we utilized an *in vitro *co-culture system consisting of 7-day old hMDMs to which we added freshly isolated autologous platelets. The use of autologous platelets excludes the possibility that platelet-macrophage interactions are the result of an immune response triggered by the recognition of platelets as 'foreign.' The hMDMs and platelets were first co-cultured in serum-free RPMI media and examined by SEM and TEM at various time points to visualize the interaction between these two different cell types. As shown in Figure 1A, we observed platelets interacting with hMDMs during the first hour of co-culture. Platelets near the macrophages became entrapped by a network of macrophage filopodia, and although the macrophages were firmly attached to the coverslip and did not migrate, they appeared to direct groups of filopodia in the direction of nearby platelets that had settled on the dish. Visualization of these cultures suggests that an interaction between human macrophages and autologous activated platelets (AAPs) occurs *in vitro*, and that it occurs in the absence of serum proteins.

**Figure 2:**
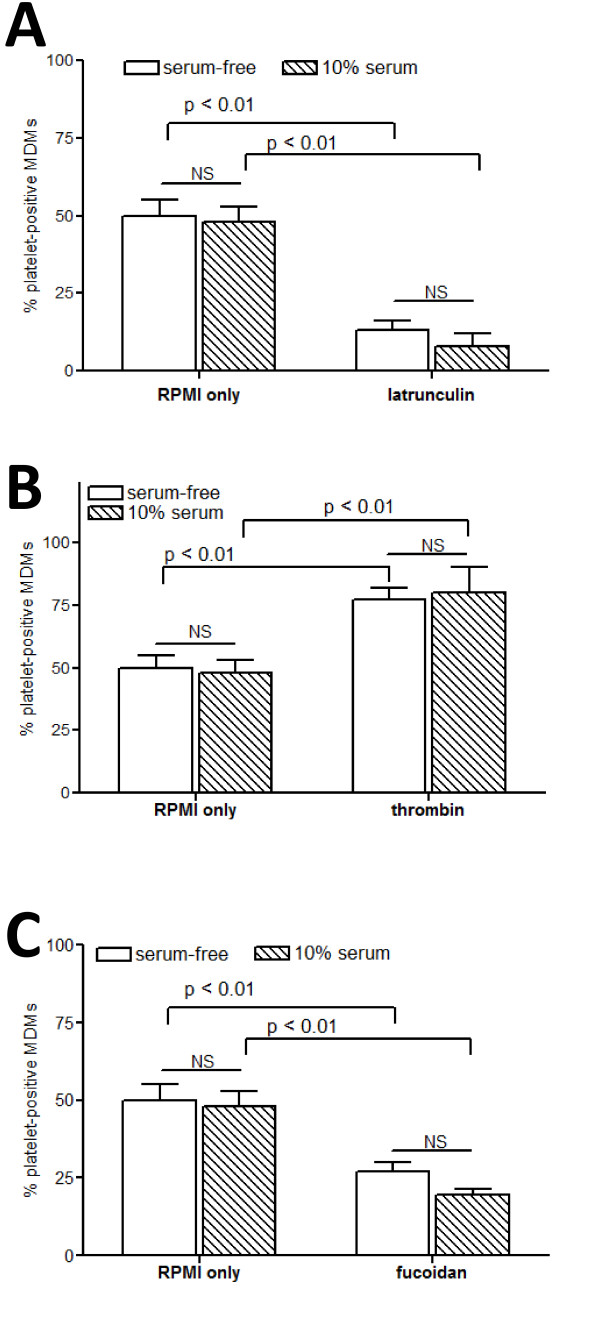
**Flow Cytometric Analysis of Platelet Phagocytosis**. hMDMs were incubated with an excess of fluorescently labeled platelets for 45 min in RPMI media alone (white bars) or containing 10% autologous human serum (striped bars). Macrophage fluorescence was measured by flow cytometry and the average percentage of FL1-positive macrophages after removal of adherent platelets are shown for 3 independent experiments for each sample. Data for control samples are repeated in each panel, and compared to treatment with **(A) **latrunculin (1 ug/ml), **(B) **thrombin (0.1 U/ml), or **(C) **fucoidan (250 ug/ml). Statistically significant differences are indicated with their corresponding p-values. The addition of 10% serum did not result in any statistically significant difference (NS).

Platelet phagocytosis by the macrophages in our co-culture system was subsequently confirmed by TEM and flow cytometry. Macrophages that were co-incubated with AAPs for one hour developed vacuoles, many of which contained contents that were the same size and shape as platelets (Figure 1B). These phagocytic vacuoles did not appear in control macrophages cultured in the absence of platelets. We then quantified phagocytosis by flow cytometric analysis of macrophage fluorescence after co-incubation with fluorescently labeled platelets and removal of adherent platelets with trypsin. When freshly isolated platelets were incubated in serum-free RPMI media and added in excess to 7-day old macrophages, approximately 50% of the macrophages internalized at least one platelet within 45 min (Figure 2). As expected, pretreatment of the hMDMs with the actin inhibitor latrunculin almost completely blocked phagocytosis, confirming the role of actin polymerization that occurs in all cases of phagocytosis (Figure 2A). The amount of phagocytosis increased if the platelets were pre-stimulated with thrombin (Figure 2B), and was significantly inhibited in the presence of fucoidan, a known competitive inhibitor to Scavenger Receptors [35,36] (Figure 2C). The presence of 10% autologous human serum had no significant effect on phagocytosis, which excludes the possibility that the platelet-macrophage interaction requires a soluble serum-bound "bridging" molecule. Together these results suggest that phagocytosis of platelets correlates with platelet activation, and that macrophage phagocytosis of autologous platelets may be mediated, at least in part, by scavenger receptors.

We also used flow cytometry to more accurately examine the level of platelet activation in different culture conditions. Platelets were analyzed for expression of P-selectin, an alpha granule component expressed during early platelet activation, and phosphatidylserine, a membrane lipid exposed on the surface of completely (and irreversibly) activated platelets. Incubation in serum-free media alone for 1 hour resulted in an approximately ten-fold increase in P-selectin expression but did not induce surface expression of phosphatidylserine (Table 1). Treatment of platelets with thrombin, known to cause complete degranulation and irreversible platelet activation [23,37], resulted in even higher levels of P-selectin and also increased surface expression of phosphatidylserine (Table 1). Interestingly, the data in Figures 1 and 2 showing phagocytosis of platelets incubated only in RPMI media suggests that only partial platelet activation, in the absence of complete degranulation or phosphatidylserine exposure, is sufficient to trigger phagocytosis. Although phagocytosis was enhanced when the platelets did express phosphatidylserine, we conclude that surface exposure of phosphatidylserine is not an absolute requirement for phagocytosis of platelets.

**Table 1:** Platelet Activation in Co-Culture Conditions

	P-selectin	Phosphatidylserine
citrated saline control	15.2 +/- 3.0	8.9 +/- 0.9

RPMI 1 hour	178.5 +/- 33.1	11.3 +/- 3.1

thrombin	1471.3 +/- 155.8	1599.9 +/- 464.5

Platelets were analyzed for platelet activation markers by flow cytometry as described in Methods. Values are expressed as Mean Fluorescence Intensity, and are the averages (+/- standard error) of at least 3 independent experiments.

### Inflammatory Cytokines are Enhanced Following Platelet Phagocytosis

The hypothesis that macrophage phagocytosis of activated platelets results in an inflammatory response that differs from the response following phagocytosis of apoptotic cells was tested by measuring the secretion of cytokines following addition of platelets or apoptotic cells to LPS-stimulated hMDMs. Autologous platelets in two different activation states were used in the co-culture experiments: platelets that were "partially activated" (with surface exposure of measurable quantities of P-selectin and CD40L) by preparing in serum-free media or "irreversibly activated" (with phosphatidylserine exposure in addition to P-selectin) by treatment with the calcium ionophore A23187 [37].

The inflammatory response of the hMDMs was assessed by measuring the levels of TNF-α, IL-6, and IL-23 after incubation with autologous primed platelets, autologous activated platelets, or control apoptotic leukocytes in the presence of LPS for 24 hours. As an additional control, we analyzed "platelets-only" cultures using the same media and incubation times as the platelet-macrophage co-cultures and were unable to detect any TNF-α, IL-6, or IL-23 in platelets alone (with or without LPS, data not shown). We therefore conclude that the cytokines secreted in this system are macrophage-derived, and in each experiment the cytokine levels were normalized to the amount of cytokine secreted by hMDMs incubated with LPS alone.

**Figure 3:**
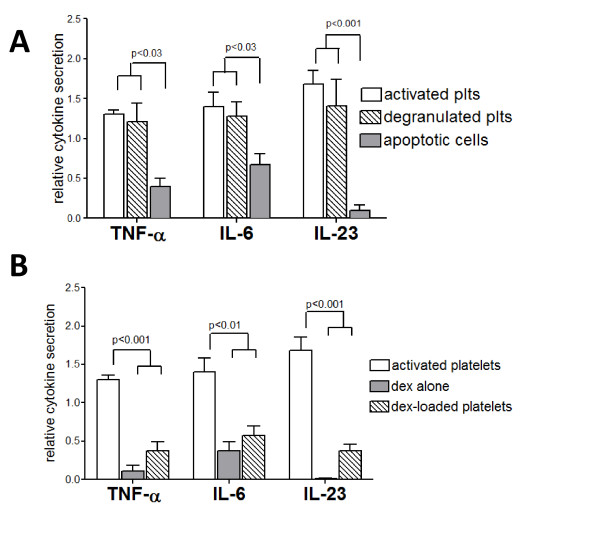
**Cytokine analysis of macrophages in the presence of activated platelets, apoptotic cells, and dexamethasone**. Cytokines were measured by ELISA 24 hrs after stimulation with LPS (100 ng/ml) in the presence or absence of **(A) **apoptotic cells or platelets, and **(B) **dexamethasone alone (1 μM) or dexamethasone-loaded platelets. Data for *activated platelets *is repeated in both panels. Cytokine levels are expressed relative to treatment with LPS only. Macrophages were incubated with equivalent numbers of platelets in each condition. Shown are the averages of at least 3 independent experiments. Statistically significant differences are indicated with corresponding p-values.

When compared to LPS stimulation alone, macrophage co-incubation with apoptotic cells inhibited LPS-induced secretion of all three pro-inflammatory cytokines (Figure 3A, grey bars). However, co-incubation with primed or activated platelets enhanced macrophage secretion of TNF-α, IL-6, and IL-23. Induction of pro-inflammatory cytokines in the presence of platelets was 20-60% higher than the levels obtained by LPS treatment alone. Furthermore, the macrophage cytokine secretion was enhanced to a similar degree after co-incubation with both partially activated and degranulated platelets (Figure 3A). These data suggest activated platelets enhance LPS-induced macrophage cytokine secretion even when they present phosphatidylserine to the macrophage.

Based on the knowledge that platelets can bind glucocorticoids via glucocorticoid receptors [38], we tested the hypothesis that glucocorticoid-bound platelets would be less inflammatory than platelets that are activated, but otherwise unmodified. Platelets were incubated with dexamethasone, then unbound glucocorticoid was removed by washing the platelets in citrated saline. Although it is unlikely that all of the dexamethasone becomes bound to the platelets, complete retention of the dexamethasone would yield a final concentration in the DEX-plt stock of 50 μM and a final (effective) concentration in the platelet-macrophage co-cultures of 1 μM. As shown in Figure 3B (striped bars), the levels of cytokines produced after co-culture with dexamethasone-loaded platelets were inhibited to 30-50% of the levels produced by stimulation with LPS alone. The dexamethasone-loaded platelets had a similar effect on cytokine secretion as 1 μM dexamethasone alone. These results indicate that the pro-inflammatory platelet effect on macrophage activation can be reversed by pre-loading the platelets with glucocorticoids.

## Discussion

Phagocytosis is an important means of clearing both immunologically compromised and apoptotic cells. Monocyte-derived macrophages are efficient phagocytes in organs of the reticuloendothelial system and within injured tissues; however, the process of platelet clearance by macrophages is poorly understood. This work has demonstrated phagocytosis of fresh autologous activated platelets (AAPs) by monocyte-derived macrophages using an entirely human-derived *in vitro *system. In this system, uptake of freshly isolated platelets is dependent on actin polymerization, but occurs independently of any soluble serum factors. Additionally, phagocytosis of platelets is enhanced with platelet activation.

Previous studies on platelet phagocytosis have focused on modified platelets such as chilled platelets, opsonized platelets, and aged platelets [39-46], each of which involves distinct changes to the platelet surface. Aged platelets most closely resemble freshly activated platelets because during aging platelets increase expression of phosphatidylserine and P-selectin [20]. Interestingly, Brown et al. have also shown *in vitro *that phagocytosis of aged platelets is mediated by scavenger receptors [20]. Therefore, macrophages may recognize freshly activated platelets in the same way that they clear aged platelets. The finding that phosphatidylserine exposure is not a requirement for phagocytosis of AAPs in the present study was somewhat unexpected, considering that phosphatidylserine is a well documented 'eat me' signal for the phagocytosis of many different types of cells undergoing apoptosis [47-50].

Because phagocytosis correlates with platelet activation, we would expect no phagocytosis to occur in the presence of quiescent platelets. However, RPMI media alone causes platelet activation (Table 1), probably due to the presence of low levels of calcium and phosphate. Additionally, the use of platelet inhibitors such as aspirin, EDTA, or prostaglandin, which may have maintained the platelets in a resting state, could not be used because they directly affect macrophage function [51,52]. Thus, one disadvantage of our system is that the platelet-macrophage interaction using activated platelets could not be compared to an interaction in which the platelets were in a truly resting state. Nonetheless, the interaction involving activated platelets is relevant because platelets are most likely activated at sites of tissue injury and perhaps during removal in the spleen. Thus, the interaction involving activated platelets was the focus of this work.

The macrophage response following phagocytosis of cells expressing surface phosphatidylserine is usually immunosuppressive [2,26,27,53,54]. In the present study, co-culture with apoptotic cells inhibited production of pro-inflammatory cytokines by LPS-activated macrophages. These results are in agreement with previous findings for TNF-α, IL-1β, IL-8, IL-12 [2,4,55,56], and are extended to now include IL-6 and IL-23.

In contrast to the effect of apoptotic cells, activated platelets enhanced pro-inflammatory cytokine secretion from LPS-activated macrophages. The cytokines measured in the current study, TNF-α, IL-6 and IL-23, are significant readouts because they are known to be secreted by macrophages, but not platelets, and they play important roles in mediating pro-inflammatory responses. Interestingly, the pro-inflammatory cytokine secretion was also enhanced by platelets with surface phosphatidylserine exposure. The finding that degranulated platelets, washed free from their secreted proteins, also enhanced LPS-induced macrophage cytokine secretion suggests that a secreted platelet factor is not likely to be responsible for this effect. However, a secreted platelet factor could exert the observed effect if it remained bound to the platelet surface after secretion from the platelet. Because the platelets remained in the co-incubation for the entire experiment (24 hrs), the possibility also exists that the inflammatory consequences of platelet-macrophage interactions occur independently of phagocytosis. Cell contact itself could be responsible for the observed effects. Nonetheless, we have shown the pro-inflammatory effect of platelets does occur in conditions which favor platelet uptake (Figure 1).

Recent studies have highlighted additional roles of platelets beyond hemostasis, particularly with respect to platelet-mediated effects on inflammation [28,57,58]. These results are particularly relevant to chronic inflammatory diseases, during which macrophages may interact with apoptotic or necrotic cells, as well as platelets, for prolonged periods of time. Studies in mice have demonstrated that depletion of platelets or platelet proteins affects macrophage infiltration and inflammation in lesions of the skin, joints, gut, and vasculature [59-63]. Although the precise mechanisms by which platelets impact macrophage activation remain unclear, the current study provides direct evidence, using human cells, of specific macrophage cytokines that are enhanced by activated platelets.

Pro-inflammatory cytokines secreted by macrophages can also exert effects on surrounding cells and tissues. For example, IL-6 and IL-23 stimulate T-cells for induction of Th17 immune responses, which are operant in autoimmune diseases such as inflammatory bowel disease, lupus, psoriasis and arthritis [64-67]. We speculate, therefore, that in addition to amplifying general pro-inflammatory responses, platelet-macrophage interactions might also play a role in Th17-mediated autoimmune diseases.

Glucocorticoids such as dexamethasone can exert powerful immunosuppressive effects on leukocytes and are thus an attractive therapy for modulating inflammation [68]. After steroid binding to glucocorticoid receptors, which occurs within the cytoplasm, activated glucocorticoid receptors translocate to the nucleus and inhibit transcription of a variety of pro-inflammatory cytokines [68]. We speculate, therefore, that the immunosuppressive action of dexamethasone-loaded platelets occurs by facilitating delivery of dexamethasone to macrophage glucocorticoid receptors. Because macrophage glucocorticoid receptors are cytoplasmic, we further speculate that the immunosuppressive effect of dex-platelets is a result of phagocytosis. The use of dexamethasone-loaded platelets for modulating macrophage action may prove useful in treating diseases characterized by excessive and unresolving inflammation. Our results demonstrating similar levels of immunosuppression with both free dexamethasone and dexamethasone bound to platelets suggests that tethering glucocorticoids to platelets may increase drug targeting and reduce the need for high systemic doses of glucocorticoids, which can have unwanted side effects [69]. Furthermore, given the role of IL-6 and IL-23 in Th17-mediated inflammatory responses, the platelet-macrophage interaction is therefore a rational pharmacological target for inhibiting some Th17-related diseases.

## Conclusions

We have shown here that the interaction of human macrophages with autologous platelets results in scavenger-receptor-mediated platelet uptake and enhancement of LPS-induced cytokine secretion. Given the presence of activated platelets together with macrophages during the response to injury and during inflammation, activated platelets at sites of inflammation most likely exacerbate the macrophage response. The presence of platelets must therefore be carefully considered when studying the cellular interactions occurring in inflammatory lesions.

We have also presented evidence here that platelets can be engineered to exert anti-inflammatory effects on macrophages. Given the emerging role of platelets in inflammatory diseases, the possibility of reversing macrophage activation with dexamethasone-loaded platelets is a promising therapeutic approach to treating unresolved inflammation.

## Competing interests

The authors declare that they have no competing interests.

## Authors' contributions

CMS conceived of the study, participated in its design and coordination, performed phagocytosis assays and cytokine measurements, and drafted the manuscript. WDH participated in the design of the study, coordinated the selection and participation of blood donors, and processed the blood samples. THF was the principal investigator on this project and provided guidance and advice on the experiments and manuscript. All authors read and approved the final manuscript.
